# Métastases endobronchiques d’un mélanome malin d’origine rectale: cas clinique et revue de la littérature

**DOI:** 10.11604/pamj.2013.14.67.1232

**Published:** 2013-02-18

**Authors:** Rachid Bouchentouf, Amine Benjelloun, Zrara Ibtissam, Moulay Ali Aitbenasser

**Affiliations:** 1 Service de pneumologie, Hôpital Militaire Avicenne, Marrakech, Maroc; 2 FMPM, Université Cadi Ayyad, Marrakech, Maroc; 3 Service d’anatomo-pathologie, Hôpital Militaire Avicenne, Marrakech, Maroc

**Keywords:** Tumeur, Mélanome malin, métastases endobronchiques, Tumour, malignant melanoma, endobronchial metastases

## Abstract

Le mélanome anorectal est une tumeur rare représentant 1,5% de tous les mélanomes et moins de1% des cancers anorectaux. Son pronostic est redoutable à cause de la survenue précoce de métastases. Le poumon est un site métastatique classique du mélanome malin, les localisations endobronchiques sont toutefois rares. Des mélanomes primitifs anorectaux ont également été décrits et sont de mauvais pronostic. Nous rapportons un cas de métastases broncho-pulmonaires de mélanome malin d’origine rectal. Comme c’est souvent le cas, les possibilités thérapeutiques sont limitées.

## Introduction

Le mélanome malin est une tumeur maligne formée à partir des cellules productrices de mélanine (mélanocytes) situées dans les couches profondes de l’épiderme. Des localisations muqueuses primitives ou secondaires ont déjà été décrites, les atteintes endobronchiques sont toutefois rares.

## Patient et observation

Nous rapportons le cas d’un patient de 52 ans, non tabagique, adressé dans notre unité pour la prise en charge d’un tableau infectieux broncho-pulmonaire évoluant depuis 2 mois associant toux grasse, dyspnée et fièvre à 38°C. L’interrogatoire retrouve la notion d’une amputation abdominopérinéale pour cancer rectal il y a 4 ans avec mise en place d’une colostomie définitive. Le compte rendu opératoire et l’examen anatomopathologique concluaient à un mélanome malin situé entre 4 et 11 cm de la marge anale. Aucun traitement complémentaire n’a été réalisé. A l’entrée, l’examen clinique retrouvait une altération de l’état général, une pâleur importante ainsi que des ronchis et des sibilants des deux champs pulmonaires.

**Figure 1:**
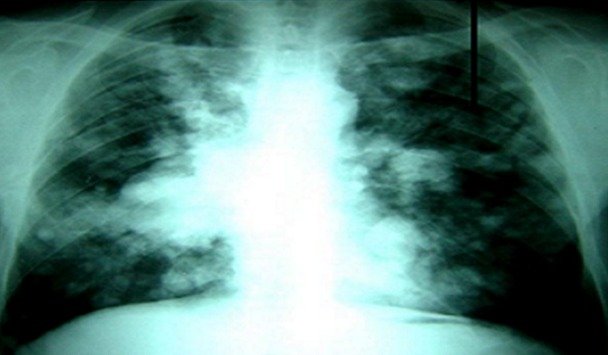
Radiographie thoracique de face montrant un aspect de lâcher de ballons bilatéral

L’examen cutané ainsi que celui des muqueuses ne retrouvait aucune lésion suspecte. Les examens biologiques montraient un syndrome inflammatoire non spécifique associé à une anémie à 8 g/dl d’Hb. La radiographie thoracique montrait un aspect de lâcher de ballons bilatéral, ainsi qu’une condensation de la base droite (Figure 1).

**Figure 2:**
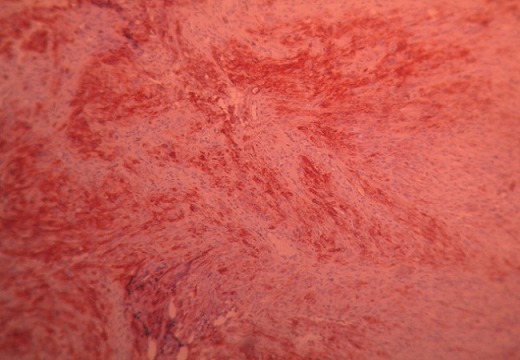
Fragment bronchique (IHCx100): marquage focal cytoplasmique fortement positif aux anticorps anti HMB 45

La bronchoscopie a montré outre une muqueuse bronchique inflammatoire, des bourgeons blanchâtres, nécrotiques et saignant au contact à l’origine de la lobaire supérieure droite et des deux pyramides basales. L’examen microscopique des différents fragments prélevés montre des faisceaux de cellules fusiformes, aux noyaux irréguliers parfois en mitose. Au sein de leur cytoplasme, existait un pigment brunâtre Perls négatif. L’immun marquage a révélé une positivité intense et diffuse aux anticorps anti HMB45 et anti PS100, en faveur d’un mélanome malin (Figure 2).

Nous avons donc posé le diagnostic de métastases broncho pulmonaires de mélanome malin d’origine rectale. En raison d’un état général altéré et du stade avancé de la maladie, le patient n’a reçu qu’un traitement symptomatique.

## Discussion

Le mélanome anorectal primitif est une tumeur rare représentant 1,5% de tous les mélanomes et moins de1% des cancers anorectaux, de pronostic redoutable à cause de la survenue précoce de métastases [1, 2] La dissémination hématogène est extrêmement fréquente et souvent précoce; 20 à 30% des patients ont des métastases au moment du diagnostic. Le foie et les poumons sont les plus touchés. Les localisations secondaires intra thoraciques sont souvent associées à des formes mutiviscérales en particulier hépatiques, osseuses voire encéphaliques [3].

Le délai d’apparition dépend de la taille des lésions initiales et peut varier de quelques semaines à plusieurs années. L’atteinte parenchymateuse isolée est la plus fréquente. Différentes lésions peuvent se voir, de manière isolée ou associée. Chen et coll. rapportent une série de 1600 patients traités pour mélanome malin de localisations diverses.

Après traitement, 260 de ces patients (16.3%) ont présenté une récidive thoracique. Les données étaient interprétables chez 130 patients, avec 52 formes multi nodulaires, 26 nodules solitaires, deux miliaires, 9 cas d’adénopathies hilaires et/ou médiastinales, 3 épanchements pleuraux, une masse extra-pleurale, un cas de lésions osseuses et 36 cas de lésions combinées [3]. D’autres auteurs ont rapporté des cas de lymphangite voire des atélectasies par obstruction bronchique ou adénopathies compressives [4, 5].

Les lésions uniques sont bien entendu de meilleur pronostic que les lésions multiples ou les atteintes osseuses [3]. Les localisations endobronchiques représentent moins de 5% des localisations thoraciques de mélanome malin. Les signes cliniques et les anomalies radiologiques sont semblables à ceux du cancer pulmonaire primitif. Les possibilités thérapeutiques demeurent très limitées. La chirurgie est réservée aux formes localisées [6]. La chimiothérapie peut être administrée seule ou en association avec l’immunothérapie dans les formes diffuses [5]. La radiothérapie externe et endobronchique peuvent être utiles dans les formes compressives ou obstructives. Les modalités de traitement sont à étudier au cas par cas. En raison des localisations multifocales fréquentes le pronostic est souvent sombre.

## Conclusion

Le mélanome malin dans sa forme métastatique peut atteindre tous les organes. En dehors des lésions dermatologiques, les formes thoraciques sont les plus fréquentes. Les atteintes endobronchiques sont toutefois rares, mais associées à un mauvais pronostic.
